# Tucumã Oil Shifted Ruminal Fermentation, Reducing Methane Production and Altering the Microbiome but Decreased Substrate Digestibility Within a RUSITEC Fed a Mixed Hay – Concentrate Diet

**DOI:** 10.3389/fmicb.2018.01647

**Published:** 2018-07-26

**Authors:** Aline F. O. Ramos, Stephanie A. Terry, Devin B. Holman, Gerhard Breves, Luiz G. R. Pereira, André G. M. Silva, Alexandre V. Chaves

**Affiliations:** ^1^Animal Science Graduate Course, Veterinary Medicine Institute, Federal University of Pará, Belém, Brazil; ^2^School of Life and Environmental Sciences, Faculty of Science, The University of Sydney, Sydney, NSW, Australia; ^3^Lacombe Research and Development Centre, Agriculture and Agri-Food Canada, Lacombe, AB, Canada; ^4^Institute of Physiology, University of Veterinary Medicine, Hanover, Germany; ^5^Embrapa Dairy Cattle, Juiz de Fora, Brazil

**Keywords:** oil supplementation, cattle, Amazonia, rumen stimulation technique, rumen microbiome

## Abstract

Tucumã oil is sourced from the fruit pulp of the tucumã tree and contains high concentrations of unsaturated fatty acids and carotenoids. Due to these properties it may have the potential to decrease enteric methane (CH_4_) from ruminants when included in the diet. The objective of this study was to determine the effect of oil mechanically extracted from the fruit pulp of tucumã on fermentation characteristics, CH_4_ production and the microbial community using the rumen stimulation technique. Treatments consisted of a control diet (forage:concentrate; 70:30), and tucumã oil included at 0.5 or 1.0% (v/v). Addition of tucumã oil linearly decreased (*P* < 0.01) dry matter disappearance. Total gas (mL/d) and carbon dioxide (CO_2_) production (mL/d, mL/g DM) were unaffected (*P* ≥ 0.36) to increasing addition of tucumã oil where 0.5% (v/v) of Tucumã oil numerically increased both variables. Acetate and butyrate percentages of total VFA were linearly decreased (*P* ≤ 0.01) and propionate and valerate percentages of total VFA were linearly increased (*P* < 0.01) by increasing concentrations of tucumã oil added to the substrate. The ratio of acetate to propionate was linearly decreased (*P* < 0.01) with increasing concentration of tucumã oil. Methane production (mL/d) was linearly decreased (*P* = 0.04) with increasing addition of tucumã oil to the substrate. Tucumã oil reduced the bacterial richness and diversity when included at 1.0% (v/v) in both solid- and liquid- associated microbes. The abundance of the genera *Fibrobacter* and *Rikenellaceae* RC9 gut group were decreased and *Pyramidobacter*, *Megasphaera*, *Anaerovibrio*, and *Selenomonas* were enriched by the addition of 1.0% tucumã oil. In conclusion, tucumã oil resulted in the favorable shift in fermentation products away from acetate toward propionate, decreasing the production of CH_4_ when tucumã oil was included at 1.0% (v/v), however, substrate digestibility was also inhibited. The rumen microbiota was also altered by the addition of tucumã oil.

## Introduction

The enteric production of methane (CH_4_) from livestock accounts for approximately 40% of total greenhouse gases (GHG) emitted from livestock production systems (Gerber et al., 2013). Brazil has the third largest cattle population in the world, making significant contributions to increasing GHGs emitted into the atmosphere. Methane emitted from cattle also constitutes as a 2–12% loss of gross energy intake (Johnson and Johnson, 1995) providing incentive for producers to decrease enteric CH_4_ production.

Enteric production of CH_4_ in ruminants is directly affected by microbial communities present in the rumen producing varing amounts of CH_4_ depending on the composition of the diet being fed (Henderson et al., 2015). Dietary supplementation with plant derived oils is considered one of the most effective methods for suppressing ruminal methanogenesis (Beauchemin et al., 2007). The mechanisms of action depend on the composition and type of fat but include depression of ciliate protozoa and methanogen populations, dilution through replacement of fermentable carbohydrates, biohydrogenation of free unsaturated fatty acids and reduction of ruminal organic matter fermentation (Eugene et al., 2008; Beauchemin et al., 2009; Knapp et al., 2014). Not only does oil supplementation provide energy to the diet, many sources of fat contain a range of secondary compounds which have the potential to further inhibit ruminal methanogenesis (Delgado et al., 2012). Fats may also be employed to alter the fatty acid composition of ruminant products (milk, meat) to improve its associated healthiness (Adeyemi et al., 2016).

Tucumã is one of the many oleaginous palms spread throughout the Amazonian region of South America. It belongs to the genus *Astrocaryum* of which 26 species are native to the southeast Amazon basin (Alexandre et al., 2015). It produces fruit in which the pulp can be pressed to extract oil for human consumption, with the dried oilseed press cake being used for animal feed (Ramos et al., 2011). Its extractivism plays an important economic role for people within these areas, especially with interest in its use as a biofuel (Bora et al., 2001). Tucumã oil is known to be rich in low molecular weight fatty acids as well as carotenoids which are naturally occurring antioxidant pigments (Bora et al., 2001). Oleic acid is the main constituent in tucumã oil extracted from the fruit pulp, followed by palmitic acid (Ferreira et al., 2008). Oleic acid is an unsaturated fatty acid, which can act as a hydrogen sink through biohydrogenation (Johnson and Johnson, 1995), however, this role in decreasing CH_4_ emissions is considered minor (Jenkins et al., 2008).

We hypothesized that the inclusion of tucumã oil would decrease CH_4_ emissions and alter the ruminal bacterial community. As such, the objective of this study was to investigate the effects of tucumã oil supplementation on *in vitro* rumen fermentation parameters, CH_4_ production and the rumen microbiota using the rumen stimulation technique (RUSITEC).

## Materials and Methods

The donor cows used in this experiment were cared for in accordance with the guidelines of the German Animal Welfare Act approved by the Lower Saxony State Office for Consumer Protection and Food Safety (LAVES, approval number AZ 33.4-42505-04-13A373).

### Experiment Design and Treatments

The experiment was conducted as a completely randomized design with three treatments duplicated in two runs with two replicates per run. The three treatments consisted of a control (no tucumã oil inclusion) and two different inclusions of tucumã oil in the substrate DM at 0.53% (0.5%) and 1.07% (1.0%) of liquid vessel volume (4 and 8 mL/d, respectively; 0.38 and 0.77 mL/g substrate DM). The experimental period was 15 days with day 1–7 used for adaptation and day 8–15 used for measurements. Tucumã oil was administered throughout the whole experimental period.

The substrate used was a hay:concentrate (70:30 DM basis; 92.3% DM, 13.5% CP, 62% NDF, and 5.2% ash – DM basis; **Table 1**) using hay obtained from natural grassland of Lower Saxony, Germany. Hay was prepared using an electrical clipper with a 76-mm blade (Duarte et al., 2017a). The commercial concentrate was pelleted (Deuka Schaffutter, Deutsche Tiernahrung Cremer, Düsseldorf, Germany) and contained 0.9% calcium, 0.55% phosphorus and 0.2% sodium. Both the hay and substrate were weighed into the same nylon bag (10 cm × 5 cm, pore size 50 ± 10 μm; *in situ* nylon bags Ankom Technology, Macedon NY, United States) for a total mass of 10.4 g of substrate (DM-basis).

**Table 1:** Chemical composition of the hay and concentrate (%DM) and fatty composition of Tucumã oil.

	Hay	Concentrate^1^
Dry matter (DM, %)	92.1	92.7
Crude protein (CP)	10.7	20.0
Neutral detergent fiber (NDF)	69.7	44.1
Ether extract (EE)	1.1	3.1
Ash	5.53	4.54
Non-fiber carbohydrates (NFC)	13.0	28.3
**Fatty acid composition**		
**Fatty acid (FA)**	**% FA in Tucumã oil**	
Palmitic (C16:0)	23–28 (SFA)	
Stearic (C18:0)	2–3 (SFA)	
Oleic (C18:1 *cis-9*)	60–68 (UFA)	
Linoleic (C18:2 ω6)	1–3 (PUFA)	
Linolenic (C18:3 ω3)	2–4 (UFA)	
n-3:n-6	0.75	
	**Total**	
Saturated fatty acids (SFA)	25.6	
Unsaturated fatty acids (UFA)	74.4	


*^*1*^The commercial concentrate was produced by Deuka Schaffutter, Deutsche Tiernahrung Cremer, Düsseldorf, Germany, 0.9% calcium, 0.55% phosphorus and 0.2% sodium. PUFA, polyunsaturated fatty acids. NFC = [100 – (CP + NDF + EE + ash)].*

The fruit of the tucumã palm (*Astrocaryum vulgare* Mart) was obtained from a closed forest at Santo Antônio do Tauá in the state of Pará, Brazil during the fruiting period. It is typical palm of the Amazon, used as food source and income of the local communities. The crude or refined oi is commercialized internationally. Fruits were sorted for quality, washed, sanitized, and then dried at room temperature in polyethylene plastic packaging. Pulp was removed from the fruit and distributed in trays to dehydrate at 40°C for 72 h. The dried pulp was then cooled at room temperature and then crushed into a fine powder, using a processor. To obtain the oil, the powder was then pressed using a commercial hydraulic press (Marconi, model ME 098/A, Piracicaba, SP, Brazil) at room temperature with the initial and final pressure at 7500 and 30,000 kg/cm^2^, respectively (Ferreira et al., 2008). After pressing, press cake (pulp) is separated from the oil. Tucumã oil has a dark orange color, rich in beta-carotene, 0.982 g/L density, melting point 27–35°C which is in the liquid form, and the saponification index of 188.4 mg KOH/g. A profile of the major fatty acids in the oil were provided by the Amazon Oil Industry (Ananindeua, Pará, Brazil) and are shown in **Table 1**.

### Inoculum Sampling and Incubation Procedure

Rumen inoculum was obtained from two ruminally cannulated Holstein cows, 2 h after morning feeding. Cattle were fed grass hay *ad libitum* and 600 g/d of a commercial concentrate. These were the same feeds used as substrate. Rumen contents were separated into rumen fluid and solid content by straining through gauze. Samples for DNA were taken from the solid and liquid fractions from each cow and frozen in liquid nitrogen (-196°C). Samples were then stored at -40°C until they were placed in a freeze dryer (over 48 h). Once dried, samples were kept at -20°C until DNA extraction.

Fluid samples from each cow were pooled together and the pH and redox potential were recorded. Samples (2 mL) were also taken and stored at -20°C for determination of volatile fatty acids (VFA; Duarte et al., 2017a) and ammonia (NH_3_-N; Duarte et al., 2017a).

Prewarmed 800 mL fermentation vessels were placed in the RUSITEC apparatus (Czerkawski and Breckenridge, 1977) and water was kept at 39°C. Each fermentation vessel had an inner vessel which contained one nylon bag with 70 g of solid digesta, and one bag containing the basal substrate in which the tucumã oil was added immediately before incubation. Tucumã oil was inserted into the bags using a pipette (4 and 8 mL for 0.5 and 1.0% (v/v) treatments, respectively). Each fermenter was filled with approximately 750 mL of rumen fluid and infused with McDougall’s buffer at a dilution rate of 30 mL/h. The inner vessels were continuously moved up and down by an electric motor to ensure adequate mixing between fluid and particles. After the first 24 h of incubation, the bag with the solid rumen digesta was replaced with a bag containing the substrate. Bags were replaced with a fresh bag containing feed after 48 h of incubation, replacing one bag per day. Bags from day 15 were not used for DM determination as they were only incubated for 24 h. Effluent was collected in 2 L glass flasks which were kept on ice to arrest bacterial growth and fermentation.

### Sample Collection

Dry matter disappearance (DMD) at 48 h was determined on day 8 and day 10–13 when bags were not used for DNA extraction. After removal from the vessel, feed bags were washed in 50 mL of pre-warmed buffer in a small plastic bag, gently squeezed and the residual buffer was placed back into the fermenter to ensure transfer of solid-phase-associated microorganisms. The residual feed bag was rinsed under cold water until the water was clear and then dried at 55°C for 48 h for the determination of DMD (Duarte et al., 2017a).

Total daily gas production was collected in gas-tight bags (Plastigas, Linde AG, München, Germany). From day 8 to 15, before measurement of total gas, two 20 mL aliquots were taken from the septum of each gas bag and transferred into evacuated tubes for the analysis of CH_4_ and carbon dioxide (CO_2_). Total daily gas production was measured using a drum-type meter (Ritter Apparatebau, Bochum, Germany).

During bag exchange, fermenter pH, gas production and effluent volume for each fermenter was measured. The pH and redox potential of the vessel was measured daily during bag exchange using a Knick pH meter (digital pH meter 646, Knick, Berlin, Germany). Effluent from each fermenter was measured and two samples (2 mL) of effluent were taken and stored at -40°C until analyzed for VFA and NH_3_-N. Ammonia and VFA data are presented as concentrations, molar percentages of total VFA as well as total production. Daily ammonia and VFA production were calculated by multiplying NH_3_-H and VFA concentrations by the effluent volume (Duarte et al., 2017a,b).

### DNA Extraction

On day 5, 10, and 15 nylon bags (solid associated microbes; SAM), as well as 30 mL of fermenter liquid (liquid associated microbes; LAM – d15 only) were removed from each vessel and immediately placed in liquid nitrogen for later extraction of DNA. Samples were taken across 3 days (e.g., day 5, 10, and 15) to evaluate differences in microbial communities across the experiment. Samples were stored at -40°C until they were placed in a freeze dryer (over 48 h). Samples were then finely ground using a coffee grinder and placed back into the freezer until DNA extraction. The liquid samples were freeze dried for 4 day and then ground using a mortar and pestle.

Total DNA was extracted from each sample using a QIAamp Fast DNA stool mini kit (Qiagen, Hilden, Germany), according to the manufacturer’s instructions. DNA yield and purity were measured using a NanoDrop spectrophotometer (ThermoFisher Scientific, Waltham, MA, United States). Extracted DNA was stored at -20°C until 16S rRNA gene library preparation and sequencing.

### Sequencing of the 16S rRNA Gene

The modified 515-F and 806-R primers as found in Walters et al. (2016) were used to PCR amplify the V4 hypervariable of both the archaeal and bacterial 16S rRNA gene. The PCR conditions and 16S rRNA gene sequencing was as previously described (Duarte et al., 2017a). Briefly, a two-step PCR was used to generate the 16S rRNA gene amplicons and these amplicons were then sequenced on an Illumina MiSeq instrument (Illumina, Inc., San Diego, CA, United States) using the MiSeq Reagent Kit v2 (500 cycles) (Illumina, Inc.), according to manufacturer’s instructions.

The 16S rRNA gene sequences were processed using the R-package DADA2 (v. 1.4) (Callahan et al., 2016) and included primer removal and truncating both the forward and reverse reads at 225 bp. The pair-end reads were then merged, and chimera sequences removed. The SILVA SSU database v. 128 (Quast et al., 2012) and the RDP naïve Bayesian classifier (Wang et al., 2007) with a 50% confidence threshold were used to assign taxonomy to each inferred 16S rRNA gene sequence; defined here as an operational taxonomic unit (OTU) with 100% sequence similarity. QIIME v. 1.9.1 (Caporaso et al., 2010) was used to measure richness (number of OTUs) and the Shannon diversity index. Bray–Curtis dissimilarities were calculated using the R packages vegan (v. 2.4.4) (Oksanen et al., 2017) and phyloseq (v. 1.20.0) (McMurdie and Holmes, 2013).

All 16S rRNA gene sequences were deposited into the NCBI Sequence Read Archive under BioProject accessions PRJNA416148.

### Chemical Composition

Feed was analyzed following the Association Official Analytical Chemistry [AOAC], 1998 method for DM (method 967.03). Neutral detergent fiber (NDF) content was analyzed according to Van Soest et al. (1991) with the use of sodium sulfite and heat-stable α-amylase. Methane and CO_2_ was measured by using gas chromatography (GC, 2014, Shimadzu Europa GmbH, Duisburg, Germany) and CH_4_ and CO_2_ production was calculated by multiplying the total gas volume by the percentage of CH_4_ with correction for temperature and pressure (0°C, 101.3 kPa; Riede et al., 2013). Determination of NH_3_-N concentrations was carried out as described previously by Riede et al. (2013). Briefly, 1 mL of sample was centrifuged at 4600 × *g* for 10 min. From the supernatant, 50 mL were mixed with 5 mL phenol solution (106 mM phenol, 0.17 mM sodium nitroprusside dehydrate) and 5 mL sodium hypochlorite solution (1% v/v sodium hypochlorite; 125 mM NaOH) and kept at room temperature for 10 min. After an incubation step at 60°C for 10 min, NH_3_-N concentration was determined photometrically at 546 nm in a spectrometer (DU 640, Beckman Coulter GmbH, Krefeld, Germany) using a NH_4_Cl standard solution (5 mM). Volatile fatty acids concentrations were analyzed by a gas chromatography system (model 5890 II, Hewlett Packard, Böblingen, Germany) equipped with a 1.8 m × 2 mm glass column packed with Chromosorb WAW (mesh 80/100) with 20% neopentyl glycol succinate and 2% orthophosphoric acid. Helium was used as a carrier gas with a flow rate of 25 mL/min. Injection port, detector and oven temperatures were 220°C, 250°C, and 130°C, respectively. The daily VFA production was estimated by multiplying VFA concentration by the volume of effluent (Duarte et al., 2017b).

### Statistical Analysis

#### Fermentation Data

The univariate procedure of SAS (SAS, Inc. 2018; SAS Online Doc 9.1.4) was used to test for normal distribution of the data. Fermentation data were analyzed using the MIXED procedure of SAS (SAS, Inc., 2018; SAS Online Doc 9.1.4). The model included the fixed effects of treatment, day and treatment × day interactions with the day of sampling (e.g., day 8–15) from each fermenter treated as a repeated measure. Therefore, the individual fermenter (*n* = 4 per treatment) was used as the experimental unit for statistical analysis (Avila-Stagno et al., 2014). The method for computing denominator degrees of freedom was Kenwardroger. The DDFM = KENWARDROGER option performs the degrees of freedom calculations detailed by Kenward and Roger (1997, 2009). The minimum values of Akaike’s information criterion were used to select the covariance structure among Compound Symmetry, Heterogeneous Compound Symmetry, Autoregressive, Heterogeneous Autoregressive, Toeplitz, Heterogeneous Toeplitz, Ante-dependence, Unstructured and Banded for each parameter. When *P* ≤ 0.05 for Type III fixed effects for treatment, orthogonal polynomial contrasts were carried out to test for linear and quadratic responses to increasing concentrations of tucumã oil [0, 0.5%, and 1.0% (v/v)]. Since there was no quadratic effect (*P* ≥ 0.05) observed in any parameter tested, these *P*-values were omitted from the tables. Treatment means were compared using the least squares mean linear hypothesis test (LSMEANS/DIFF). Significance was declared at *P* ≤ 0.05 and tendencies 0.05 < *P* ≤ 0.10.

#### Microbiome Data

All samples were randomly subsampled to 44,500 sequences prior to analysis to account for differences in sequencing depth. The number of OTUs per sample and the Shannon index were compared by treatment group using a linear mixed model in R v. 3.4.2 (R Core Team, 2017) using lme4 v. 1.1.12 (Bates et al., 2014) with fermenter as the random effect and tucumã oil treatment, and sampling time (e.g., day 5, 10, and 15) as fixed effects, followed by Tukey’s Honest Significant Difference (Lenth, 2016). Both the archaeal and bacterial community structure was analyzed using permutational multivariate analysis of variance (PERMANOVA) and the Adonis function with 10,000 permutations in vegan. The betadisper function in vegan was used to assess the homogeneity of dispersion for each time point. Linear discriminant analysis (LDA) effect size (LEfSe) (Segata et al., 2011) was used to identify genera with a relative abundance of greater than 0.1% that were differentially abundant between the control and 1% tucumã oil treatments for both LAM and SAM samples at day 15. A minimum LDA score of 4.0 was used as the threshold for classifying differentially abundant genera.

## Results

### Effect of Tucumã Oil on *in Vitro* Fermentation

Dry matter disappearance was linearly decreased (*P* < 0.01) with increasing addition of tucumã oil to the substrate (**Table 2**). Acetate and butyrate percentages of total VFA (mmol/100 mmol) linearly decreased (*P* ≤ 0.01) and propionate and valerate percentages of the total VFA were linearly increased (*P* < 0.01) with increasing concentration of tucumã oil. Consequently, the ratio of acetate to propionate (*P* < 0.01) linearly decreased with increasing addition of tucumã oil to the substrate. In agreement with the molar percentages of VFA data (**Table 2**), propionate production (mmol/d) tended (*P* = 0.08) to increase, and butyrate and valerate production (mmol/d) increased (*P* ≤ 0.05) with increasing concentration of tucumã oil (**Supplementary Table S1**). There was a significant interaction between treatment and day for acetate production (mmol/d), where 1% tucumã supplementation decreased (*P* < 0.01) acetate production at days 10 and 12–14 compared to control (interaction means not shown). There was also a significant treatment by day interaction for ammonia production (mmol/d), where 1% tucumã supplementation decreased ammonia production compared to the control at day 10 only (interaction means not shown)

**Table 2:** Effect of Tucumã oil on dry matter digestibility, pH, redox, the molar percentages of individual volatile fatty acids (VFA), ammonia and total VFA concentrations in a Rusitec fed a mixed hay – concentrate diet.

	Concentration of Tucumã Oil (v/v)			SEM	*P*-value				Covariance structure
				
	Control (*n* = 4)	0.5% (*n* = 4)	1% (*n* = 4)		Treatment	Day	Treatment × Day	Linear	
Dry matter disappearance (%)	46.1a	39.9ab	34.4b	2.31	0.02	0.22	0.76	<0.01	Autoregressive
pH	6.79	6.77	6.79	0.020	0.67	<0.02	0.99	0.99	Heterogeneous Autoregressive
Redox	-236.9	-230.6	-229.1	5.76	0.61	<0.01	0.91	0.36	Heterogeneous Toeplitz
Total VFA (mmol)	27.2	28.4	24.9	2.49	0.60	0.12	0.41	0.53	Ante-dependence
Acetate (A; mmol/100 mmol)	52.2a	45.0b	37.7c	1.18	<0.01	0.34	0.37	<0.01	Ante-dependence
Propionate (P; mmol/100 mmol)	27.1c	32.2b	37.2a	1.24	<0.01	<0.01	0.31	<0.01	Heterogeneous Autoregressive
Butyrate (mmol/100 mmol)	14.9a	13.2b	12.2b	0.51	0.01	0.16	0.50	<0.01	Ante-dependence
Valerate (mmol/100 mmol)	3.28c	6.14b	9.44a	0.703	<0.01	0.07	0.18	<0.01	Unstructured
BCVFA (mmol/100 mmol)	2.46	3.30	3.44	0.984	0.76	0.26	0.87	0.50	Toeplitz
A:P	1.93a	1.40b	1.01b	0.069	<0.01	<0.01	0.45	<0.01	Heterogeneous Autoregressive
NH_3_-N (mmol/L)	6.53	6.70	6.24	0.191	0.26	<0.01	0.35	0.14	Autoregressive
Effluent (mL/d)	697	692	683	16.5	0.84	0.04	0.45	0.56	Heterogeneous Toeplitz


*VFA, volatile fatty acids. BCVHA, branched-chain VFA (iso-valerate + iso-butyrate). Different letters in rows indicate significantly different means (*P* < 0.05).*

Total gas production (mL/d) was unaffected (*P* = 0.45) by tucumã oil supplementation (**Table 3**). Similarly, tucumã oil addition did not affect (*P* ≥ 0.36) production of CO_2_ (mL/d, mg/g DM, mg/g DMD). Methane production (mL/d) was linearly decreased (*P* = 0.05) where production at 1% tucumã oil was halved compared to the control and 0.5% inclusion of tucumã oil (**Table 3**). When CH_4_ was expressed as mg CH_4_/g DM and mg CH_4_/g DM disappeared, treatment × day interaction was significant. Only on day 12 and 15, mg CH_4_/g DM was lower in treatment 1% tucumã compared to the control. For mg CH_4_/g DM disappeared, CH_4_ was only different on day 12 and 13 within the 0.5% (v/v) treatment, with no other differences observed between treatments.

**Table 3:** Effect of Tucumã oil on gas production in a Rusitec fed a mixed hay – concentrate diet.

	Concentration of Tucumã Oil (v/v)			SEM	*P*-value				Covariance structure
				
	Control (*n* = 4)	0.5% (*n* = 4)	1% (*n* = 4)		Treatment	Day	Treatment × Day	Linear	
Total gas (mL/d)	784	878	734	75.7	0.45	0.08	0.55	0.64	Toeplitz
CO_2_ (mL/d)	57.1	76.4	51.5	12.31	0.36	0.53	0.32	0.75	Heterogeneous Toeplitz
CO_2_ (mg/g DM)	10.8	14.5	9.8	2.33	0.36	0.53	0.31	0.76	Heterogeneous Toeplitz
CO_2_ (mg/g DM disappeared)	25.5	35.5	29.2	5.28	0.43	0.97	0.89	0.64	Compound Symmetry
CH_4_ (mL/d)	16.5a	16.1a	8.3b	5.88	0.05	0.01	0.55	0.04	Ante-dependence
CH_4_ (mg/g DM)	1.23	1.36	0.57	0.367	0.35	<0.01	<0.01	<0.01	Toeplitz
CH_4_ (mg/g DM disappeared)	2.8	3.2	1.5	1.04	0.50	0.57	0.03	0.38	Toeplitz


*Different letters in rows indicate significantly different means (*P* < 0.05).*

### Effect of Tucumã Oil on the Rumen Microbiota

The LAM and SAM samples had significantly different bacterial community structures (*R*^2^ = 0.08; *P* ≤ 0.001); however, most of the abundant genera were shared between the two sample types (**Supplementary Figures S1, S2**). This included *Prevotella*, *Megasphaera*, *Anaerovibrio*, *Rikenellaceae* RC9 gut group, *Fibrobacter*, *Lactobacillus*, and *Selenomonas*.

**FIGURE 1:**
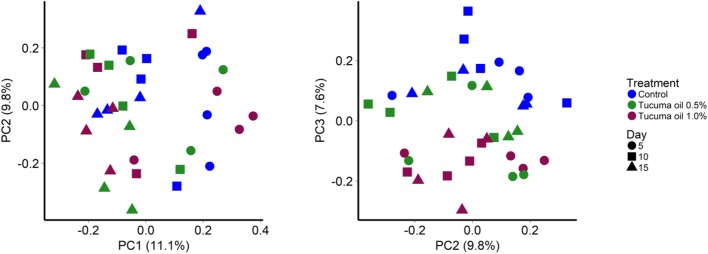
Principal coordinates analysis plots of the Bray-Curtis dissimilarities for solid-associated microbes (SAM) by treatment and sampling time. Percentages of variation explained by the principal coordinates are indicated on the axes.

Among SAM samples, sampling time had a slightly larger effect (**Figure 1**; *R*^2^ = 0.099; *P* < 0.001) than tucumã oil supplementation on the structure of the rumen microbial community (*R*^2^ = 0.089; *P* < 0.001). The greatest effect of tucumã treatment was observed at day 15 (**Supplementary Figures S3, S4**; *R*^2^ = 0.240; *P* = 0.007) as the control samples were most dissimilar from both tucumã oil treatment samples at this point (**Supplementary Figure S3**).

**FIGURE 2:**
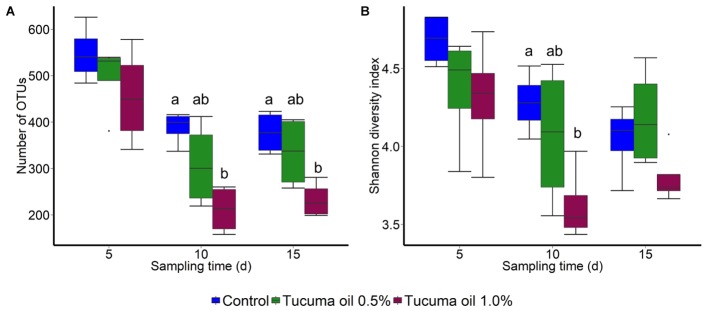
Box plots of the **(A)** Number of OTUs and **(B)** Shannon diversity index by treatment and sampling time for solid-associated microbes (SAM). Different lowercase letters within each sampling time indicate significantly different means (*P* < 0.05) *n* = 4.

**FIGURE 3:**
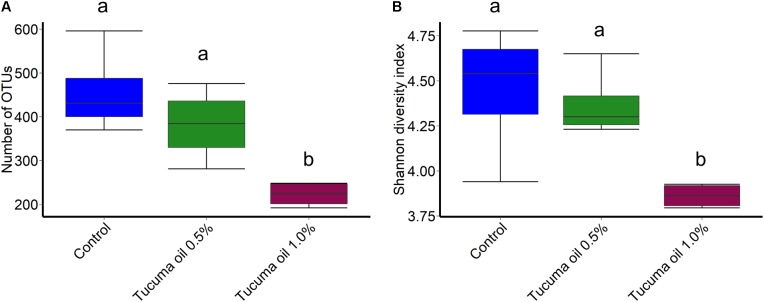
Box plots of the **(A)** number of OTUs and **(B)** Shannon diversity index by treatment for liquid-associated microbes (LAM; day 15). Different lowercase letters indicate significantly different means (*P* < 0.05).

**FIGURE 4:**
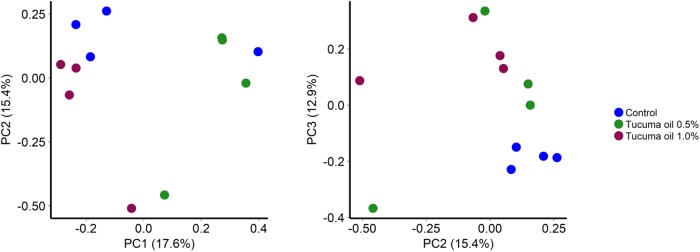
Principal coordinates analysis plots of the Bray-Curtis dissimilarities for liquid-associated microbes (LAM) samples by treatment at day 15. Percentages of variation explained by the principal coordinates are indicated on the axes *n* = 4 (*P* < 0.05; LDA score ≥ 4.0).

Tucumã oil also affected the richness (number of OTUs) and diversity (Shannon index) of the ruminal microbiota. The number of OTUs was significantly decreased in the 1.0% tucumã oil samples at day 10 and day 15 for SAM (**Figure 2A**) and day 15 for LAM (**Figure 3A**) compared with the control treatment. The Shannon diversity index was also lower in the 1.0% tucumã oil LAM samples compared with both the 0.5% tucumã oil and control treatment at day 15 (**Figure 3B**). Among the SAM samples, only at day 10 did the 1.0% tucumã treatment significantly affect the Shannon diversity index (**Figure 2B**). The addition of tucumã oil also had a comparable effect in the LAM samples (day 15) (**Figure 4**; *R*^2^ = 0.250; *P* = 0.006). In fact, the three treatment groups were more dissimilar to each other in terms of Bray–Curtis dissimilarities than at any sampling time for the LAM samples (**Supplementary Figure S5**).

Given that the greatest dissimilarity among samples was observed at day 15 between the 1.0% tucumã oil and control treatments, we identified genera that were most strongly associated with each treatment group at day 15 for both LAM and SAM samples (LDA score > 4.0; **Table 4**). Many of the differentially abundant genera between the two treatments were the same for both LAM and SAM samples including; *Fibrobacter*, *Rikenellaceae* RC9 gut group, *Megasphaera*, *Selenomonas*, *Anaerovibrio*, and *Pyramidobacter*. Tucumã oil appeared to have the greatest positive effect on *Olsenella* (33.6-fold increase) and *Pyramidobacter* (23.5-fold increase) in the LAM samples.

**Table 4:** Differentially abundant genera identified between the control and 1% Tucumã oil treatment for liquid- and solid- associated microbes (LAM and SAM, respectively) in a RUSITEC system with a mixed hay – concentrate diet.

Genus	LDA score	*P*-value	Relative abundance (%)	
			
LAM samples			Control	Tucumã oil 1.0% (v/v)
**Control**		
*Fibrobacter*	4.50	0.0209	6.52 ± 1.75	0.38 ± 0.10
*Rikenellaceae* RC9 gut group	4.49	0.0209	6.18 ± 0.35	0.55 ± 0.10
**Tucumã oil 1% (v/v)**		
*Pyramidobacter*	4.63	0.0209	0.38 ± 0.05	8.94 ± 0.82
*Megasphaera*	4.54	0.0433	7.29 ± 1.02	14.2 ± 2.31
*Phocaeicola*	4.47	0.0209	0.91 ± 0.27	6.73 ± 1.52
*Anaerovibrio*	4.34	0.0209	0.83 ± 0.23	5.16 ± 0.68
*Selenomonas*	4.18	0.0209	0.83 ± 0.08	3.70 ± 0.35
*Acidaminococcus*	4.12	0.0209	0.15 ± 0.09	2.55 ± 0.60
*Olsenella*	4.04	0.0209	0.06 ± 0.03	2.03 ± 0.74
**SAM samples**		
**Control**		
*Fibrobacter*	4.77	0.0209	14.49 ± 3.01	2.52 ± 0.62
*Lachnospiraceae* XPB10140 group	4.12	0.0209	2.55 ± 0.10	0.24 ± 0.16
*Rikenellaceae* RC9 gut group	4.09	0.0202	2.71 ± 0.21	0.42 ± 0.07
**Tucumã oil 1% (v/v)**		
*Megasphaera*	4.65	0.0209	11.26 ± 1.98	21.19 ± 3.07
*Prevotella*	4.58	0.0209	10.35 ± 1.18	17.19 ± 1.11
*Selenomonas*	4.20	0.0209	1.25 ± 0.12	4.41 ± 0.22
*Anaerovibrio*	4.09	0.0209	0.31 ± 0.04	2.69 ± 0.41
*Pyramidobacter*	4.01	0.0209	0.27 ± 0.04	1.97 ± 0.22


*Relative abundance values represent mean percent ± standard error of the mean. LDA = linear discriminant analysis.*

Only three methanogenic genera were detected including *Methanobrevibacter*, *Methanosphaera*, and *Methanomicrobium*. There was no effect on the relative abundance on any of the detected methanogenic genera in either LAM and SAM samples at any sampling time (**Supplementary Table S2**).

## Discussion

Tucumã oil was included at 0.50 and 1.0% (v/v) of total fermenter volume due to the dilution factor of the oil in the RUSITEC. Duarte et al. (2017b) suggested when they included Pequi oil into a Rusitec fermenter at 6% dietary fat content of the TMR, that when expressed as the total fermenter volume, oil was only included at 0.18% of total fermenter volume. It was suggested that this may have contributed to conflicting results between the Rusitec and a previous *in vitro* batch culture they had conducted. Whilst necessary for *in vitro* experiments, this expression of treatment is not extrapolable to the rumen diet as when calculated on a DM basis these concentrations constitute as 27.4 and 43.0% of total added substrate (i.e., TMR and Tucumã oil).

Dry matter disappearance was decreased by up to 25.6% in the 1.0% tucumã oil treatment compared to the control substrate which is not a desirable effect. This reduction in DM disappearance coincides with a decrease in the richness and diversity of the ruminal microbiota on day 10 and 15. *Fibrobacter*, a genus of fibrolytic bacteria were also lower in relative abundance in the 1.0% tucumã oil treatment which is consistent with the negative effect that oils have on fiber-digesting microbes within the rumen (McIntosh et al., 2003). The relative abundance of *Rikenellaceae* RC9 gut group was also negatively affected by tucumã oil. Similarly, Zened et al. (2013) found that the abundance of *Rikenellaceae* RC9 gut group was decreased by 79% in the rumen of cattle fed a diet supplemented with 5% DM sunflower oil. *Rikenellaceae* is a relatively new bacterial family and therefore its metabolic function in the rumen has not yet been defined; however, Pitta et al. (2010) suggested that members of this family are associated with either the primary or secondary degradation of carbohydrates as its abundance was decreased as increased proportions of bermuda grass were substituted for wheat in a cattle diet.

Tucumã oil has been evaluated for its anti-inflammatory properties due to its high carotenoid content and has been shown to effectively improve the immune system in mice (Baldissera et al., 2017). The Tucumã plant also possesses chemical antioxidant compounds which could be involved in the decrease in the relative abundance of *Fibrobacter*, *Rikenellaceae* RC9 gut group and *Lachnospiraceae*. Similarly, Jobim et al. (2014) indicated that tucumã extracts presented antimicrobial activities against gram positive bacteria including *Enterococcus faecalis, Bacillus cereus*, and *Listeria monocytogenes* and this was related to a combination of secondary compounds including polyphenols and carotenes and their ability to disrupt redox reactions (Jobim et al., 2014).

Oleic acid is the main fatty acid constituent in tucumã oil followed by palmitic, linolenic, stearic and linoleic acid. Oleic acid is a monounsaturated omega-9 fatty acid and is found in a variety of oils including olive, canola, peanut, and high oleic sunflower and safflower oils (He et al., 2012). The hydrogenation of unsaturated fatty acids from oil is suggested to contribute to the decrease in the ability of microorganisms to saturate fatty acids, which causes unsaturated fatty acids to accumulate and interfere with microbial digestion. *Selenomonas* spp., which were enriched in the 1.0% tucumã oil treatment, can hydrate oleic acid resulting in enhanced growth for members of this genus (Maczulak et al., 1981; Hudson et al., 1995). Oleic acid can decrease ruminal CH_4_ production *in vitro* (Wu et al., 2016); however, as with the present study, there have also been reductions in DM degradability observed when oils high in oleic acids have been fed to cattle (Hristov et al., 2011). This may be related to the inhibitory effect of a variety of oils on fibrolytic bacteria (Enjalbert et al., 2017). Wu et al. (2016) demonstrated that total gas and CH_4_ production were decreased when oleic acid was included at up to 60 mg/50 mL culture solution in a batch fermentation system. In the current study, CH_4_ (mL/d) was decreased by 49.7% in the 1.0% tucumã oil treatment compared to the control; however, this was in part a result of an undesirable decrease in DM disappearance. Despite the change in CH_4_ production (mL/d), there was no effect on the relative abundance of any the methanogenic genera (**Supplementary Table S2**).

The decrease in CH_4_ production was associated with shifts in rumen fermentation favoring the production of propionate over acetate. It is well established that an increase in the ratio of propionate to acetate favors a decrease in the production of CH_4_. This is because propionate acts as alternative hydrogen sinks which decreases the availability of hydrogen to CH_4_ producing Archaea, known as methanogens (McAllister and Newbold, 2008). The observed increase in propionate production is consistent with an increase in the abundance of *Anaerovibrio* in both LAM and SAM samples as Hobson et al. (1981) indicated that these bacteria play an important role in lipolytic activity in sheep, producing glycerol for propionate synthesis (Jenkins et al., 2008). The relative abundance of *Anaerovibrio* was also increased by pequi oil in a RUSITEC study, oil which also has a high oleic acid content (Duarte et al., 2017b). Despite the change in CH_4_ production (mL/d), there was no effect on the relative abundance of any the methanogenic genera identified in this study, that is, *Methanobrevibacter*, *Methanosphaera*, and *Methanomicrobium* (**Supplementary Table S2**). However, although the PCR primers used in the present study target both the archaeal and bacterial 16S rRNA gene, they are not specific for the methanogenic populations and therefore it is possible that their abundance is underestimated in the present study.

Interestingly, *Pyramidobacter* spp., which belong to the *Synergistetes* phylum were increased by 23.5 fold in LAM and 7.5 fold in SAM samples from the 1.0% tucumã oil treatment. These bacteria are obligatory anaerobic, produce major amounts of acetic acid, and are associated with increased fiber digestion (Downes et al., 2009; Pan et al., 2017). They have also been known to increase in abundance in high CH_4_ emitting cattle (Wallace et al., 2015). This contradicts the observed decrease in the proportion of acetic acid and DM disappearance when 1.0% of tucumã oil was included in the substrate, and it remains unclear as to why this group of bacteria was increased as a result of Tucumã oil supplementation. *Prevotella* which are involved in the metabolism of proteins and peptides in the rumen (Wallace et al., 1997), were also increased in the SAM samples. *Prevotella* spp. also produce propionate (Russell and Hespell, 1981; McCabe et al., 2015) and this is consistent with the increase in propionic acid production with increasing addition of tucumã oil.

The addition of tucumã oil also decreased the production of butyrate (**Supplementary Table S1**). Butyrate production in the bovine rumen is associated with certain members of the bacterial and protozoal community (Demeyer and Van Nevel, 1979; Carberry et al., 2012). The protozoa are positively linked with an increase in CH_4_ production as the principal fermentation end products of protozoal activity are acetate and butyrate, and these two VFA provide H_2_ for methanogenesis (McAllister and Newbold, 2008). The reduction in butyrate production was simultaneous with an increase in valerate production and both VFA have competitive metabolic pathways (Kristensen et al., 2000; Kristensen and Harmon, 2004). Many members of the *Lachnospiraceae* family are major producers of butyrate (Meehan and Beiko, 2014) as evidenced by the lower relative abundance of *Lachnospiraceae* XPB1014 group in the SAM samples from the 1.0% tucumã oil treatment. The concentration of NH_3_-N was not affected when tucumã oil was included.

## Conclusion

The addition of tucumã oil resulted in the favorable shift in fermentation products away from acetate toward propionate, decreasing the production of CH_4_ when tucumã oil was included at 1.0%. However, dry matter digestibility was also negatively affected by tucumã oil addition into the substrate which is undesirable. The structure of the rumen microbiota was significantly altered and the bacterial richness and diversity decreased in the 1.0% tucumã oil treatment for both LAM and SAM samples. Tucumã oil lowered the abundance of the genera *Fibrobacter* and *Rikenellaceae* RC9 gut group and enriched *Pyramidobacter*, *Megasphaera*, *Anaerovibrio*, and *Selenomonas*. Whilst tucumã oil may be used in the ruminant diet to suppress CH_4_ production, it needs to be further evaluated using *in vitro* techniques at lower concentrations to investigate whether diet digestibility can be affected.

## Author Contributions

AC, GB, and AR: study design. AR, ST, and AC: conducting RUSITEC study. AR, ST, DH, GB, and AC: lab analysis. AR and ST: DNA extraction. DH: bioinformatics. ST, AR, DH, GB, and AC: writing the manuscript. ST, AR, DH, GB, LP, AS, and AC: manuscript revision. AS: paying for publication.

## Conflict of Interest Statement

The authors declare that the research was conducted in the absence of any commercial or financial relationships that could be construed as a potential conflict of interest.

## Supplementary Material

FIGURE S1 Relative abundance (%) of the ten most abundant genera in the solid-associated microbe (SAM) samples by treatment and sampling time.

FIGURE S2 Relative abundance (%) of the ten most abundant genera in the liquid-associated microbe (LAM) samples by treatment at day 15.

FIGURE S3 Principal coordinates analysis plot of the Bray–Curtis dissimilarities for SAM samples by treatment at day 15. Percentages of variation explained by the principal coordinates are indicated on the axes.

FIGURE S4 Bray–Curtis dissimilarities between treatments by sampling time for SAM samples. Error bars represent standard error of the mean (*n* = 16 comparisons). Different lowercase letters within each sampling time indicate significantly different means (*P* < 0.05).

FIGURE S5 Bray–Curtis dissimilarities between treatmentsfor LAM samples. Error bars represent standard error of the mean (*n* = 16 comparisons).

TABLE S1 Effect of tucumã oil on volatile fatty acids and ammonia production in a RUSITEC fed a mixed hay – concentrate diet.

TABLE S2 Percent relative abundance of methanogenic genera detected in LAM and SAM samples by sampling time and treatment (*n* = 4). ND = not detected.
