# Supplementary figures and images for: Tucumã Oil Shifted Ruminal Fermentation, Reducing Methane Production and Altering the Microbiome but Decreased Substrate Digestibility Within a RUSITEC Fed a Mixed Hay – Concentrate Diet
Source: Front Microbiol. 2018 Jul 26;9:1647. doi: 10.3389/fmicb.2018.01647 (PMC6071481; doi:10.3389/fmicb.2018.01647)

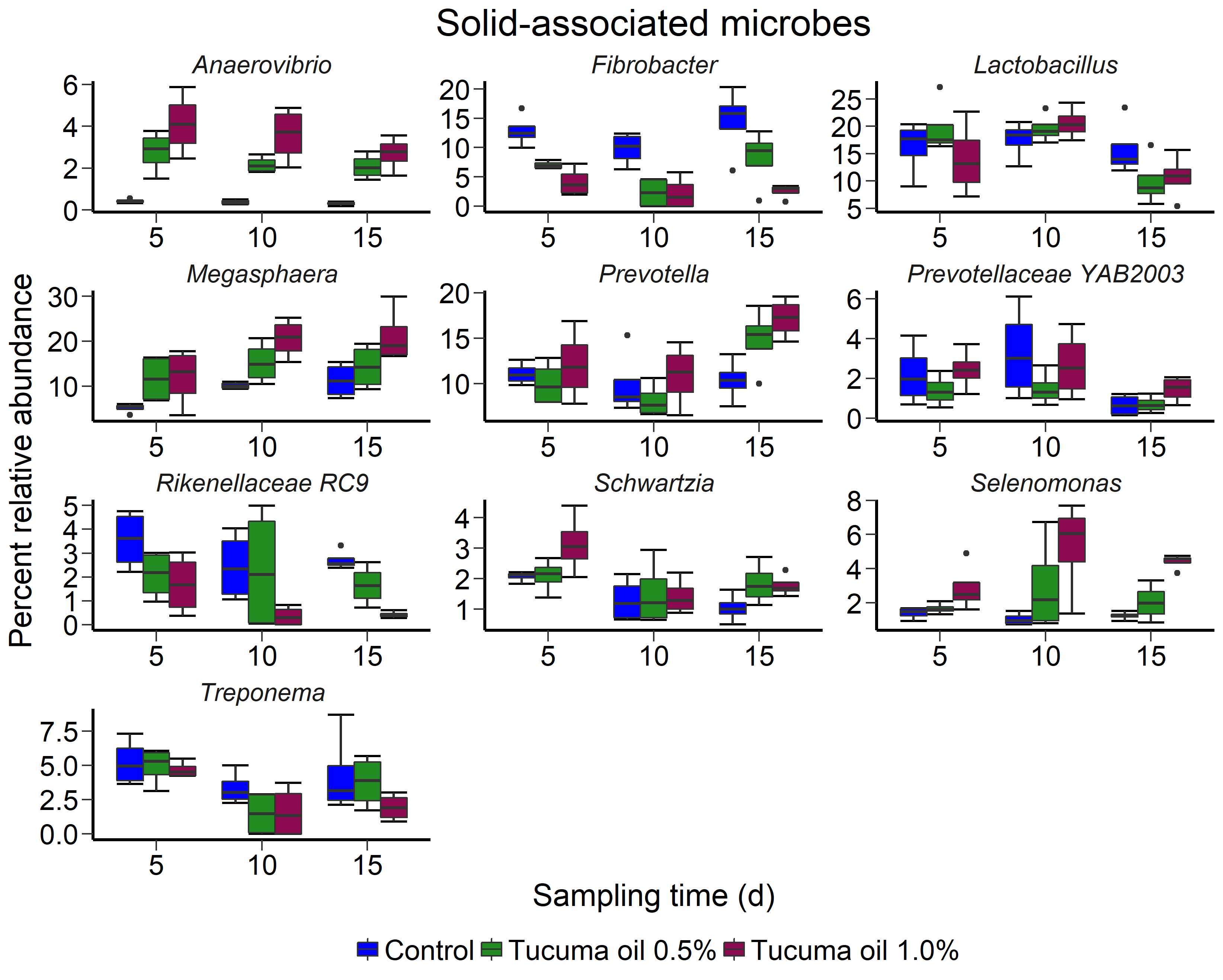

Supplement: FIGURE S1 — Relative abundance (%) of the ten most abundant genera in the solid-associated microbe (SAM) samples by treatment and sampling time. [file Image_1.TIFF]

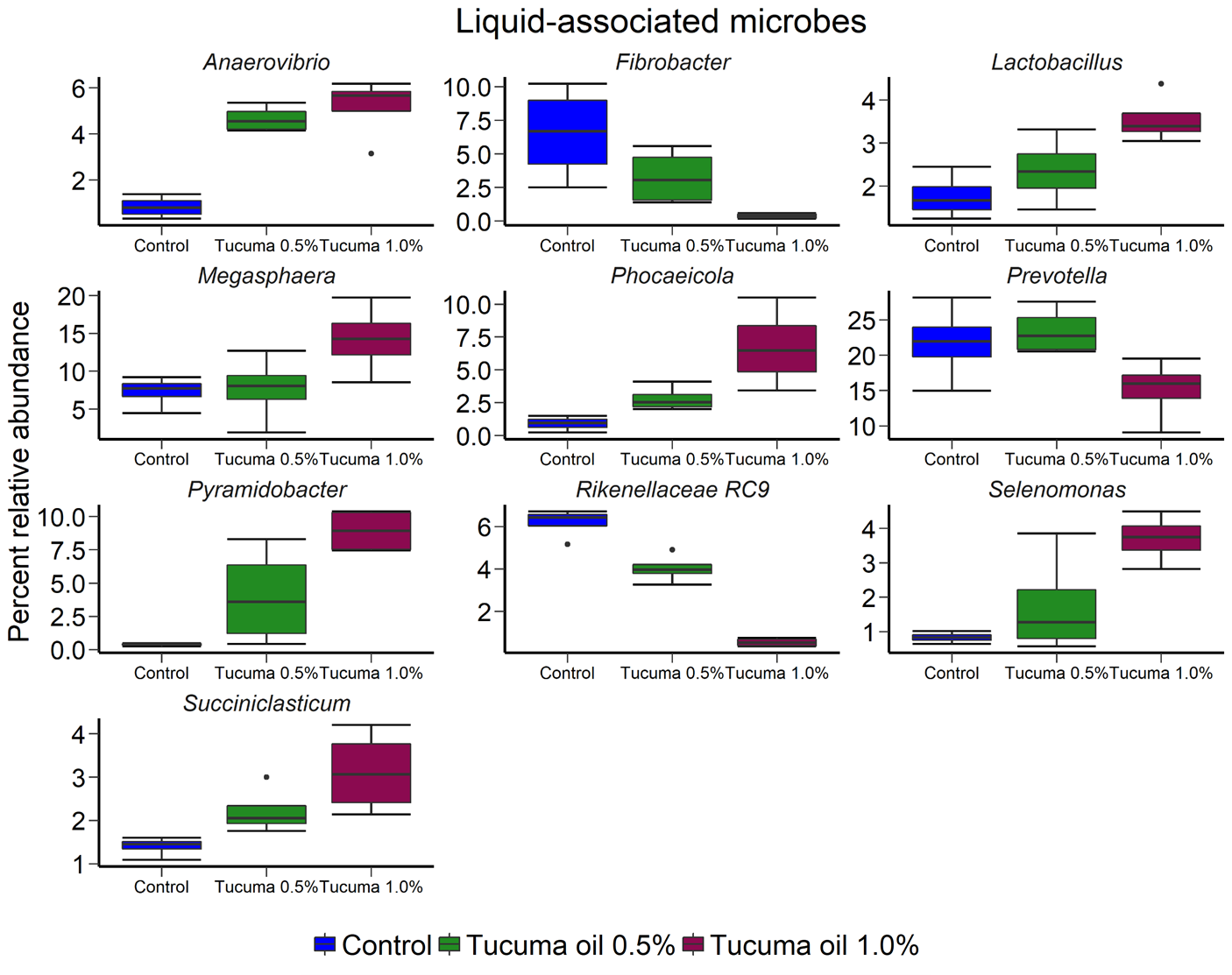

Supplement: FIGURE S2 — Relative abundance (%) of the ten most abundant genera in the liquid-associated microbe (LAM) samples by treatment at day 15. [file Image_2.TIFF]

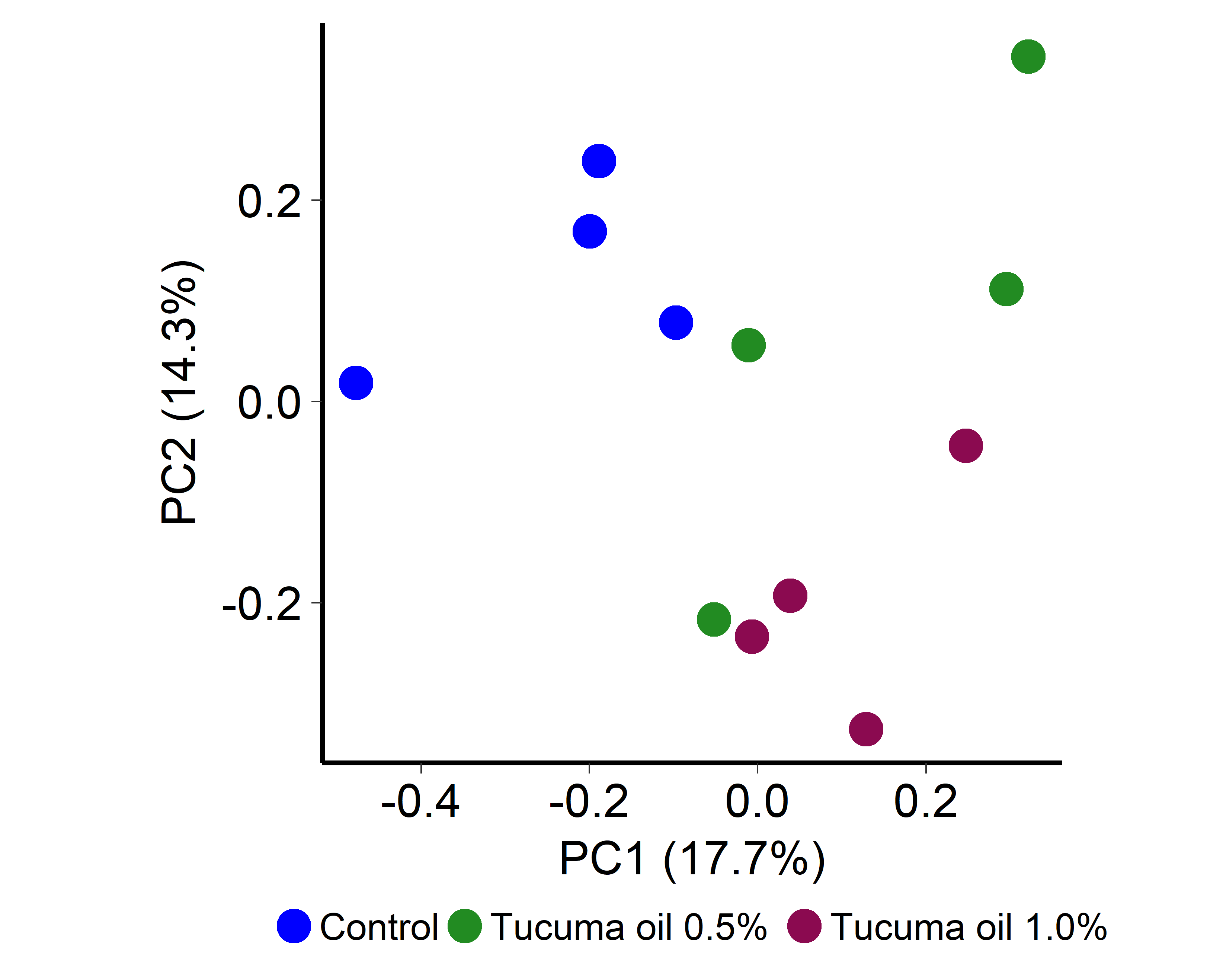

Supplement: FIGURE S3 — Principal coordinates analysis plot of the Bray–Curtis dissimilarities for SAM samples by treatment at day 15. Percentages of variation explained by the principal coordinates are indicated on the axes. [file Image_3.TIFF]

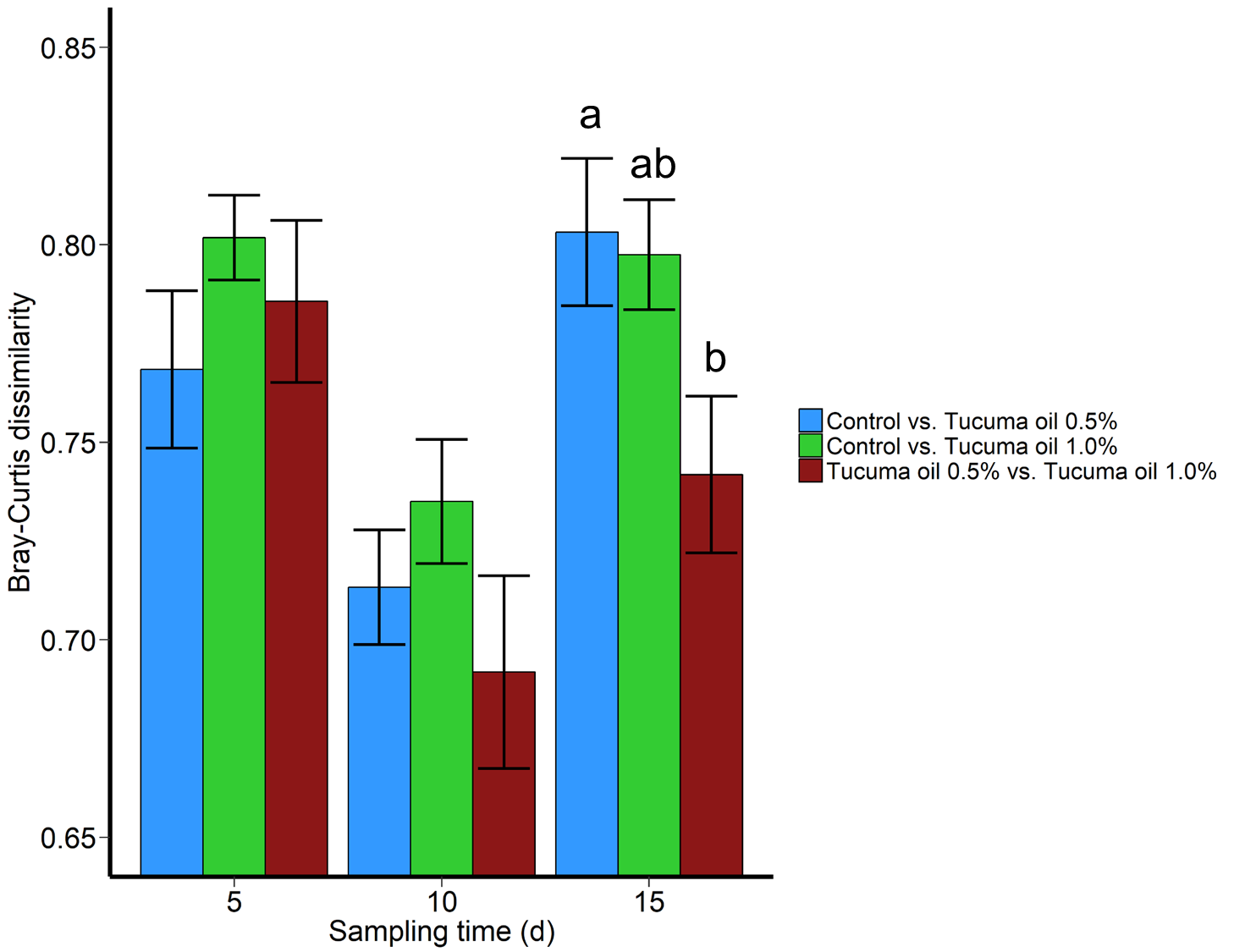

Supplement: FIGURE S4 — Bray–Curtis dissimilarities between treatments by sampling time for SAM samples. Error bars represent standard error of the mean (n = 16 comparisons). Different lowercase letters within each sampling time indicate significantly different means (P < 0.05). [file Image_4.TIFF]

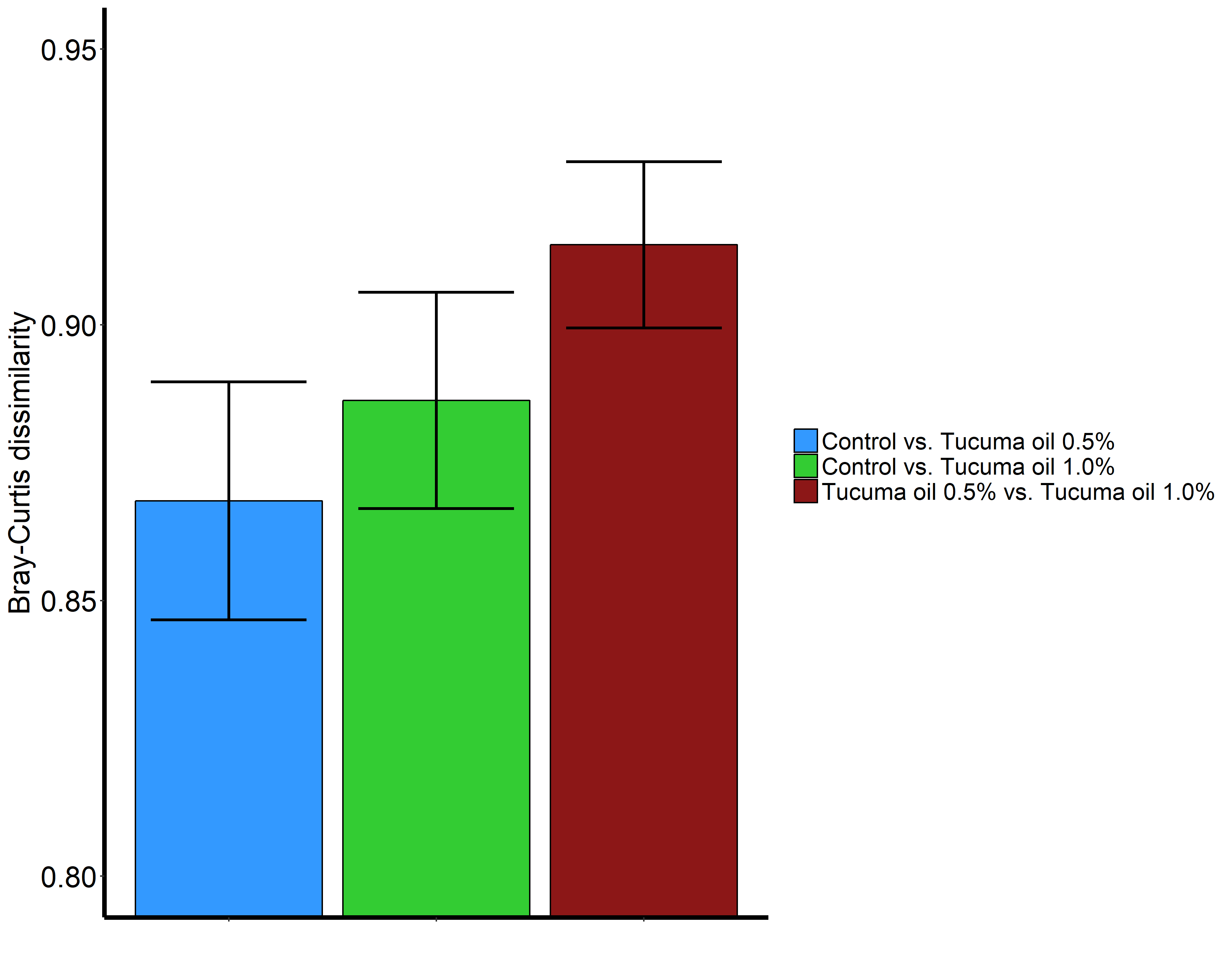

Supplement: FIGURE S5 — Bray–Curtis dissimilarities between treatmentsfor LAM samples. Error bars represent standard error of the mean (n = 16 comparisons). [file Image_5.TIFF]
