# Supplementary material for: Tucumã Oil Shifted Ruminal Fermentation, Reducing Methane Production and Altering the Microbiome but Decreased Substrate Digestibility Within a RUSITEC Fed a Mixed Hay – Concentrate Diet
Source: Front Microbiol. 2018 Jul 26;9:1647. doi: 10.3389/fmicb.2018.01647 (PMC6071481; doi:10.3389/fmicb.2018.01647)
Supplement: TABLE S1 — Effect of tucumã oil on volatile fatty acids and ammonia production in a RUSITEC fed a mixed hay – concentrate diet. [file Table_1.docx]

| **Supplementary Table S1.** Effect of Tucumã oil on volatile fatty acids and ammonia production in a Rusitec fed a mixed hay – concentrate diet. | | | | | | | | | |
| --- | --- | --- | --- | --- | --- | --- | --- | --- | --- |
|  | **Concentration of Tucumã Oil (v/v)** | | |  | ***P* value** | | | |  |
|  | **Control (*n*=4)** | **0.5% (*n*=4)** | **1% (*n*=4)** | **SEM** | **Treatment** | **Day** | **Treatment × Day** | **Linear** | **Covariance structure** |
| Total VFA (mmol/d) | 18.7 | 19.6 | 17.0 | 1.69 | 0.55 | 0.01 | 0.11 | 0.49 | Heterogeneous Toeplitz |
| Acetate (A; mmol/d) | 9.8 | 8.9 | 6.4 | 0.99 | <0.01 | <0.01 | <0.01 | 0.04 | Toeplitz |
| Propionate (P; mmol/d) | 5.1 | 6.3 | 6.2 | 0.36 | 0.08 | 0.04 | 0.43 | 0.05 | Heterogeneous Autoregressive |
| Butyrate (mmol/d) | 2.76a | 2.56ab | 2.08b | 0.177 | 0.05 | 0.14 | 0.30 | 0.02 | Ante-dependence |
| Valerate (mmol/d) | 0.61b | 1.20ab | 1.63a | 0.200 | 0.02 | 0.07 | 0.60 | 0.01 | Toeplitz |
| BCVFA (mmol/d) | 0.48 | 0.68 | 0.62 | 0.190 | 0.76 | 0.07 | 0.50 | 0.49 | Toeplitz |
| NH_3_-N (mmol/d) | 4.47 | 4.64 | 4.27 | 0.096 | 0.04 | <0.01 | <0.01 | 0.14 | Autoregressive |

Different letters in rows indicate significantly different means (*P* < 0.05).

**Supplementary Table S2.** Percent relative abundance of methanogenic genera detected in LAM (liquid-associated microbe) and SAM (solid-associated microbe) samples by sampling time and treatment (*n* = 4). ND = not detected.

|  | ***Methanobrevibacter*** | ***Methanosphaera*** | ***Methanomicrobium*** |
| --- | --- | --- | --- |
| **SAM** |  |  |  |
| **Day 5** |  |  |  |
| Control | 0.45 ± 0.10 | 0.01 ± 0.01 | 0.41 ± 0.15 |
| Tucumã oil 0.5% | 0.72 ± 0.28 | 0.02 ± 0.01 | 0.26 ± 0.09 |
| Tucumã oil 1.0% | 0.60 ± 0.23 | 0.03 ± 0.01 | 0.22 ± 0.09 |
| **Day 10** |  |  |  |
| Control | 0.38 ± 0.11 | ND | 0.15 ± 0.08 |
| Tucumã oil 0.5% | 0.67 ± 0.17 | 0.03 ± 0.01 | 0.02 ± 0.01 |
| Tucumã oil 1.0% | 0.28 ± 0.15 | 0.03 ± 0.02 | < 0.01 |
| **Day 15** |  |  |  |
| Control | 0.42 ± 0.17 | ND | 0.17 ± 0.10 |
| Tucumã oil 0.5% | 0.67 ± 0.11 | 0.01 ± 0.01 | 0.03 ± 0.02 |
| Tucumã oil 1.0% | 0.48 ± 0.16 | 0.01 ± 0.01 | 0.05 ± 0.03 |
| **LAM** |  |  |  |
| **Day 15** |  |  |  |
| Control | 0.47 ± 0.05 | ND | 0.13 ± 0.07 |
| Tucumã oil 0.5% | 0.66 ± 0.04 | 0.01 ± 0.01 | 0.04 ± 0.03 |
| Tucumã oil 1.0% | 0.51 ± 0.07 | 0.03 ± 0.02 | 0.07 ± 0.04 |

No significant differences (*P* > 0.05) were detected among treatments or sampling times for any detected genera.
